# Naloxone Use and Outcomes in Prehospital and Emergency Department Care

**DOI:** 10.7759/cureus.104074

**Published:** 2026-02-22

**Authors:** Nicholas T Ellingwood, Mark McGraw, Kavish Chandra

**Affiliations:** 1 Emergency Medicine, Dalhousie University, Saint John, CAN; 2 Emergency Medicine, Dalhousie Medicine New Brunswick, Saint John, CAN; 3 Emergency Medicine, Horizon Health Network, Saint John, CAN

**Keywords:** emergency medicine, naloxone, opioid agonist therapy, opioid toxicity, prehospital care, toxicology

## Abstract

Background

Opioid toxicity remains a major public health concern in Canada, which is driven by potent synthetic opioids such as fentanyl. Naloxone is the antidote for opioid toxicity and can be given in a variety of routes and dosages. Naloxone is administered by bystanders, first responders, emergency medical services (EMS), and emergency physicians. Despite its widespread use, there are limited local data on its administration in the prehospital and emergency department (ED) settings, as well as on its associated outcomes.

Materials and methods

This was a retrospective review study of patients presenting to a tertiary care ED in 2024 who had a discharge diagnostic code “poison narcotics” and received naloxone. Prehospital and ED charts were reviewed to collect demographics, naloxone dose, route, number of doses, reversal success, disposition, and opioid agonist therapy (OAT) initiation and referral. Descriptive statistics were used, and Fisher’s exact test was used to compare outcomes between single and multiple naloxone doses.

Results

Thirty-three patients met the inclusion criteria (mean age 40.4 years; 33.3% female). Naloxone was administered prehospital in 93.9% of cases, with 60.6% receiving naloxone prior to EMS arrival. Prehospital reversal success was 83.9%. EMS administered naloxone to 54.5% of patients at various dosages and routes. Seven patients required naloxone in the ED, and two needed an ICU admission. There were no deaths. Only 6.25% of patients were started on or referred for OAT, none directly following opioid toxicity reversal. There was no significant difference in reversal success or disposition between those who received single versus multiple doses of naloxone.

Conclusions

Most cases were successfully reversed prior to ED arrival, and many were managed by first responders and bystanders, highlighting the importance of public education and access to intranasal naloxone. Low rates of OAT initiation underscore the need to prevent barriers to treatment following an ED visit.

## Introduction

Opioid overdoses continue to be a major public health concern on the provincial and federal levels. In Canada, the opioid epidemic was declared a national public health crisis in 2016 [1]. Since then, there have been over 45,000 opioid toxicity-related hospitalizations and over 187,000 opioid toxicity-related emergency department (ED) visits in Canada [2]. The increasing prevalence of potent opioids, most notably fentanyl, has increased the number of unintentional overdoses in the past decade. Fentanyl is a synthetic opioid that is approximately 100 times more potent than morphine and is often used as an adulterant in other drugs such as heroin, cocaine, and ecstasy. In 2016, only 40% of unintentional opioid toxicity deaths involved fentanyl, whereas in 2024, 79% of unintentional opioid toxicity deaths involved fentanyl [2].

Naloxone is the first-line treatment for opioid toxicity. It is a potent opioid antagonist that can rapidly reverse the effects of opioids on all opioid receptors, including the mu receptor. The mu receptor is associated with opioid-induced respiratory depression, the main cause of death in opioid toxicity patients [3-5]. Naloxone can be administered intravenously (IV), intramuscularly, subcutaneously, and intranasally and is given at a wide range of doses based on the status of the patient, experience of the administrator, and whether the patient is opioid-tolerant or not. Clinical response is based on the patient’s Glasgow Coma Scale (GCS) and respiratory rate. Repeat dosing may be required due to rebound toxicity, as many opioids have a longer half-life than naloxone (30-90 minutes) [6]. Treatment of opioid toxicity has become more difficult with the introduction of ultra-potent synthetic opioids, which often require multiple doses to achieve successful reversal [7,8].

Given these factors, it is no surprise that we see a wide range of practice patterns among ED physicians and prehospital providers, and knowledge gaps certainly exist [9]. One study from Wyoming in 2019 found that while >95% of advanced emergency medicine technicians and paramedics were confident in administering naloxone, almost one in four (22.5%) incorrectly identified at least one use of naloxone [10]. This highlights the importance of analyzing patterns of naloxone use in the prehospital and ED settings to identify knowledge gaps and prescribing variation.

New Brunswick’s Department of Health has established surveillance mechanisms to collect and analyze prehospital data from Ambulance New Brunswick, substance toxicity deaths from the Chief Coroner’s Office, and opioid-related hospitalizations from hospital data, which are published in quarterly reports [11]. In 2023, there were 102 opioid-related hospitalizations in New Brunswick, but it is unclear what their indication for admission was, which would include naloxone infusion, ventilatory support, or monitoring.

While these reports accurately illustrate the significance of the opioid epidemic in New Brunswick, this study seeks to build on current work by quantifying naloxone use in the province. The objective of this study was to examine prehospital and ED factors associated with naloxone administration in patients presenting with opioid toxicity and to identify predictors of patient outcomes and disposition. Additionally, this study explored rates of opioid agonist therapy (OAT) initiation or addiction medicine referral among patients presenting with opioid toxicity.

## Materials and methods

Patient identification

**Figure 1:**
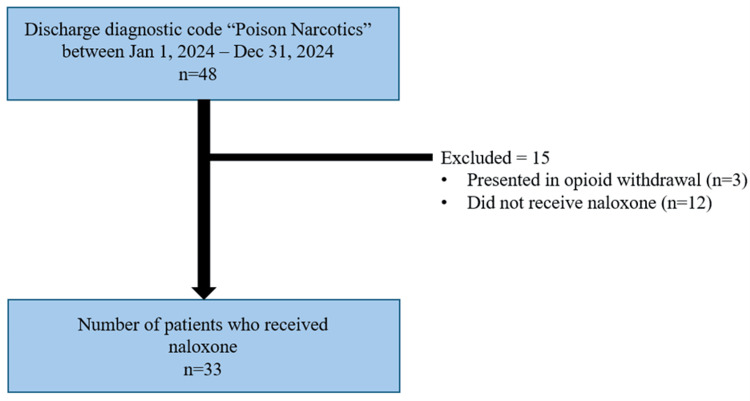
Flow diagram of patient selection process and exclusion criteria

The protocol was approved by Horizon Health Network's Research Health Board (approval number: 102187). To collect the data, charts were pulled from the Saint John Regional Hospital ED Health Records based on the following criteria: the patient presented to the ED from January 1, 2024, to December 31, 2024, had a discharge diagnostic code of “poison narcotics” (ICD-10 T40.x), and received naloxone either prehospital or in the ED, which is outlined in Figure 1. Both the ED and emergency medical services (EMS) charts for each patient were used for data collection and analyzed retrospectively. The principal investigator was the sole chart reviewer, and a standardized data collection form was used. SPSS Statistics (IBM Corp., IBM SPSS Statistics for Windows. Armonk, NY: IBM Corp.) was used for statistical analysis.

From the EMS chart, the patient's age, gender, and the date and time of EMS arrival were collected. Pre-EMS naloxone data were collected using the EMS chart, including the route, dose, number of doses, and who gave the naloxone. Based on the patient's initial EMS vitals and clinical assessment, pre-EMS naloxone administration was classified as successful or unsuccessful. Successful reversal was defined as a respiratory rate greater than 8 breaths per minute, an improvement in GCS, or no longer requiring ventilatory support. From the EMS chart, the route, dose, and number of doses were collected for all naloxone doses administered by EMS, along with pre-naloxone and post-naloxone vitals to determine whether naloxone led to a successful reversal. Whether intubation was performed prehospital was also recorded.

From the ED chart, pre- and post-naloxone vitals, the route and dose of each naloxone dose, including the naloxone infusion, were recorded. Outcomes such as successful reversal, in-hospital intubation, and disposition were all collected.

To determine whether the patient was started on or referred to start OAT, the ED chart was reviewed for buprenorphine/naloxone administration or a prescription. The ED visit was looked at on the electronic medical record to confirm if any buprenorphine/naloxone was administered and if a referral was sent to a physician who practices addiction medicine to initiate OAT.

Data analysis

Continuous variables are presented using the mean and standard deviation, or the median and interquartile range, as appropriate. Categorical variables are presented using frequencies and percentages. All naloxone doses were documented as per the EMS chart, the physician charting, and the nursing notes. Naloxone infusions were counted as a single naloxone dose even if the infusion rate changed. All patients who received a naloxone infusion had already received multiple doses; therefore, this did not affect the statistical analysis. Given the small sample size, Fisher’s exact test was used to assess for statistical significance in reversal success and disposition between those who received a single dose and those who received multiple doses of naloxone.

## Results

A total of 48 patient visits were reviewed; 33 met our inclusion criteria and were included in the analysis. The mean age was 40.4 years (range: 13 to 73, SD = 15.4). One-third of patients were female. Twenty (60.6%) patients received naloxone prior to EMS arrival, with 55% (11/20) receiving naloxone by either the Fire or Police department. Nine patients received naloxone prior to EMS arrival from members of the public, including family members, friends, bystanders, and shelter staff. Demographics and outcomes are shown in Table 1. Prior to EMS arrival, five (25%) patients received one dose of naloxone, nine (45%) received two doses, and six (30%) received three doses. Eighteen (94.7%) patients received intranasal (IN) naloxone, while one patient was administered intramuscular (IM) naloxone from a friend. The dose and route of naloxone were not specified for one patient. Of the 20 patients who received naloxone before EMS arrival, 13 (65%) had a successful reversal and did not require any naloxone by EMS or in the ED. Of the seven patients who received naloxone pre-EMS without reversal, all received naloxone from EMS, and six (85.7%) had successful reversal prior to arriving at the ED.

**Table 1:** Patient demographics and outcomes (n = 33) SD: standard deviation, EMS: emergency medical services, ED: emergency department, ICU: intensive care unit, n: number of observations, IN: intranasal, IM: intramuscular, IV: intravenous

Variable	Outcome
Age, mean (SD)	40.4 (15.4)
Sex	
Female	11 (33.3)
Male	22 (66.7)
First responder/bystander naloxone, % yes	20 (60.6)
1 dose	5 (25.0)
2 doses	9 (45.0)
3 doses	6 (30.0)
Route of administration, % IN (n = 19)	18 (94.7)
Reversal, % successful	13 (65.0)
Naloxone provider	
Fire or police department	11 (55.0)
Public	7 (35.0)
Other (e.g., staff)	2 (10.0)
EMS naloxone, % yes	18 (54.5)
1 dose	12 (66.7)
2 doses	5 (27.8)
3 doses	1 (5.6)
Route of administration, % IM (n = 18)	13 (72.2)
Prehospital reversal, % successful, n = 31	26 (83.9)
EMS intubation, % intubated	0 (0.0)
ED naloxone, % yes	7 (21.2)
1 dose	2 (28.6)
2 doses	1 (14.3)
3 doses	1 (14.3)
4 doses	2 (28.6)
7 doses	1 (14.3)
Route of administration, % IV (n = 7)	7 (100.0)
ED reversal, % successful	7 (100.0)
ED intubation, % intubated	1 (3.0)
Outcomes	
Total number of doses	
1 dose	9 (27.3)
2 doses	12 (36.4)
3 doses	6 (18.2)
4 doses	2 (6.1)
5 doses	1 (3.0)
6 doses	2 (6.1)
12 doses	1 (3.0)
Disposition from ED	
Discharged	28 (84.8)
Medicine	3 (9.1)
ICU	2 (6.1)
Location of first naloxone dose, % prehospital	31 (93.9)

**Figure 2:**
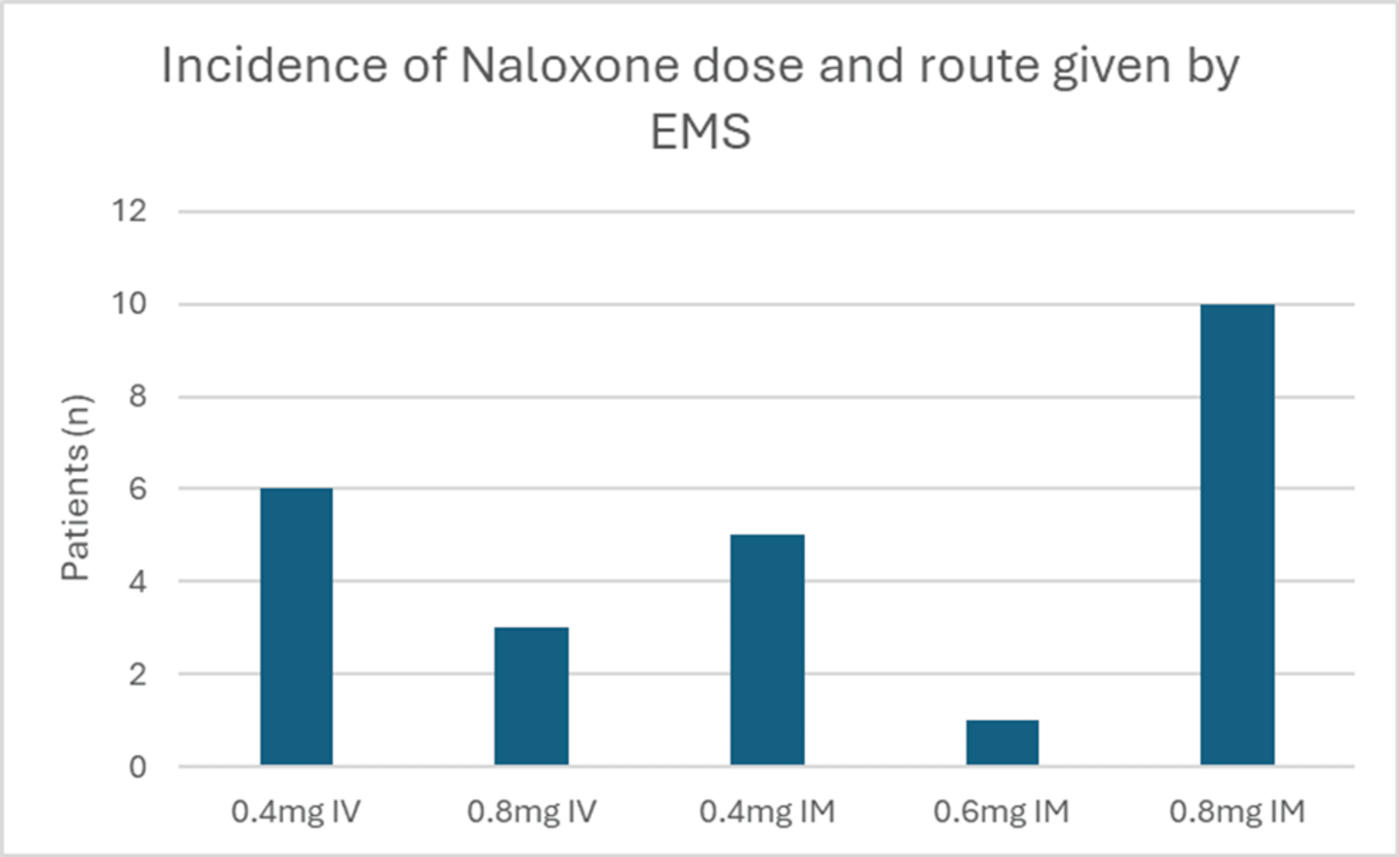
Incidence of dose and route of naloxone given by EMS EMS: emergency medical services, IV: intravenous, IM: intramuscular

Eighteen of the 33 patients received naloxone from paramedics, which is summarized in Figure 2. Thirteen (72.2%) of those patients received IM naloxone, whereas five (27.8%) received IV naloxone. Paramedics administered a total of 16 IM naloxone doses: 0.4 mg was given five times (31.5%), 0.6 mg was given one time (6.3%), and 0.8 mg was given 10 times (62.5%). Paramedics administered a total of nine IV doses. Six (66.7%) of these were 0.4 mg, and three (33.3%) were 0.8 mg. Of the 18 patients who received naloxone by paramedics, 12 received a single dose, and 10 had successful reversal. Meanwhile, six of them received multiple doses of naloxone, and three of those had successful reversal. In all, 93.9% (31/33) of patients received naloxone prior to ED arrival, with 83.9% (26/31) achieving successful reversal. There were no out-of-hospital intubations.

A total of seven patients received naloxone in the ED. Two of those did not receive any prehospital naloxone. Both received a single dose of naloxone 0.4 mg IV and had successful reversal and were discharged. A total of 23 doses of naloxone were given in the ED, all administered IV, with a range of 0.04-0.8 mg; infusions counted as a single dose. Naloxone infusions were started on three patients, two of whom were stopped in the ED, and one continued in the intensive care unit (ICU). All seven patients had successful reversals. One intubation was performed in the ED, but the patient was subsequently extubated and discharged. Of the seven patients who received naloxone in the ED, three were discharged, three were admitted to the floor (medicine/pediatrics), and one was admitted to the ICU. One patient who received prehospital naloxone was admitted to the ICU for a reason unrelated to opioid toxicity.

There was no significant difference in pre-EMS reversal success between those who received a single dose of naloxone and those who received multiple doses, as illustrated in Table 2 (p = 0.521). There was no significant difference in reversal success between those who received a single dose or multiple doses of prehospital naloxone (p = 0.562). There was no significant difference in discharge disposition between those who received one dose of naloxone and those who received multiple doses (p = 0.574).

**Table 2:** Comparison of patient outcomes with the number of naloxone doses given ^a^Fisher's exact test, EMS: emergency medical services, ED: emergency department, ICU: intensive care unit, NC: not calculable

	One dose	More than one dose	p-value^a^
Pre-EMS reversal success			0.521
No	0 (0.0)	7 (38.9)	
Yes	2 (100.0)	11 (61.1)	
Prehospital naloxone reversal success			0.562
No	0 (0.0)	5 (20.8)	
Yes	7 (100.0)	19 (79.2)	
ED reversal success			NC
No	0 (0.0)	0 (0.0)	
Yes	2 (100.0)	5 (100.0)	
EMS intubation required			NC
No	9 (100.0)	24 (100.0)	
Yes	0 (0.0)	0 (0.0)	
ED intubation required			1.000
No	9 (100.0)	23 (95.8)	
Yes	0 (0.0)	1 (4.2)	
Discharge disposition			0.574
Medicine/pediatrics	0 (0.0)	3 (12.5)	
ICU	0 (0.0)	2 (8.3)	
Discharged	9 (100.0)	19 (79.2)	

A total of 48 charts with the discharge diagnostic code “poison narcotics” were reviewed to see if the patients were started on OAT regardless of whether they received naloxone or not. Of those 48 patients, three were started on OAT or referred to an addiction medicine specialist. Two did not receive any naloxone and were started on buprenorphine/naloxone after presenting to the ED in opioid withdrawal. The last patient was started on OAT as an inpatient. There were no instances of patients being referred to an addiction medicine specialist from the ED after presenting with opioid toxicity and requiring naloxone.

## Discussion

Interpretation

Our study had 33 patients who presented to the ED in opioid toxicity, with a large portion of patients having successful reversal prior to arrival to the ED (83.9%), and a very low percentage required admission to the ICU (6.1%). No patients died. Taken together, our results highlight that many patients with opioid toxicity were initially managed by non-EMS personnel, including members of the public, and overall, they had high rates of successful reversal.

Previous studies

Our findings reinforce the importance of public health interventions, public education, and easily accessible take-home naloxone kits. Two different studies found very high rates of survival from opioid toxicity when take-home naloxone kits were used. In British Columbia, Canada, between 2012 and 2018, Moustaqim-Barrette et al. found a survival rate of 98.1% from opioid toxicity when a take-home naloxone kit was used [12]. A systematic review by McDonald and Strang found a survival rate of 99.1% with take-home naloxone [13]. However, these two studies included only take-home naloxone, not naloxone administered by the police and fire department prior to EMS arrival. Their findings of high success with pre-EMS naloxone are consistent with this study, where there was a high reversal success with pre-EMS naloxone (65%) with no instances of death. A key finding in this study is that it illustrates the importance of public education surrounding naloxone administration with opioid toxicity and ensuring adequate access to take-home kits for at-risk populations and those around them.

In our study, there was variation in practice patterns among EMS providers regarding naloxone route and dose, as illustrated in Figure 1. Although this study is not large enough to determine whether different routes and dosing affect the rate of reversal success and outcomes, it does demonstrate that further research is needed to determine the optimal route and dose for EMS providers. It highlights the importance of a standardized approach to opioid toxicity management. Tylleskar et al. looked at naloxone administration by EMS in Norway between 2014 and 2018 [14]. They found that IM naloxone was administered 91.9% of the time, while 1.9% received IV naloxone, and 3.8% received a combination of IM and IV naloxone. The proportion of patients receiving IV naloxone by EMS was much higher in our study. The contrast between Tylleskar et al. and this study raises the question of whether EMS providers are delaying naloxone administration by prioritizing vascular access or if there is a difference in training.

One confounding variable in this study is the potential for polysubstance use. A combination of opioids with other sedative medications increases the risk of respiratory depression. If a sedative such as hypnotics, alcohol, or benzodiazepines were used in conjunction with opioids, then we would expect naloxone to have less of an effect, which could lead to more doses of naloxone and higher rates of admission. The rate of polysubstance use was not examined in this study, but there are increasing rates across Canada. Konefal et al. found that the proportion of polysubstance deaths among toxicity deaths was 57% in 2017 compared to just 39% in 2014 [15]. The high rate of polysubstance use likely inflates the percentage of patients with opioid toxicity who require admission due to poor response to naloxone.

Clinical and research implications

While not a primary objective of our study, we demonstrated that a very low percentage of patients were initiated or referred to start OAT, which occurred only in patients who presented with opioid withdrawal. There were no instances where a patient presented in opioid toxicity requiring naloxone and then subsequently was referred to an addiction medicine physician to initiate OAT. It is unclear in this study whether this is due to emergency physicians not discussing OAT with patients or to patients declining a referral. These interactions with the healthcare system are a crucial opportunity to see if they would like to be referred to an addiction medicine specialist or initiate OAT. Hu et al. looked at the rate of OAT initiation within seven days of an ED visit for opioid toxicity between 2013 and 2020 for patients with opioid use disorder who were not admitted [16]. They studied over 20,000 visits and found that OAT was initiated within seven days 4.1% of the time. Although the initiation rate was low, these findings underscore the importance of emergency physicians routinely discussing OAT with patients following opioid toxicity.

Limitations

A limitation of this study is the small sample size. With only 33 patients, we lacked adequate power to detect statistical significance in comparing outcomes between those who received a single dose of naloxone and those who received multiple doses. Although this study lacks power, it does identify the frequency at which opioid toxicity is being managed successfully prior to arrival in the ED and the naloxone administration patterns among EMS providers. Another limitation in this study is that the patients were selected solely based on discharge diagnostic code, meaning some patients who also presented with opioid toxicity with different diagnostic codes would be missed. This results in selection bias because emergency physicians are probably more likely to use the discharge diagnostic code “poison narcotics” if they had a positive response to naloxone. For example, this study likely misses a cohort of patients with opioid toxicity who present to the ED in cardiac arrest or with a hypoxic brain injury from opioid toxicity. These patients would have a higher rate of ICU admission and mortality and could have different discharge diagnostic codes.

## Conclusions

Opioid toxicity continues to be a growing concern across Canada. It is becoming more challenging to treat, given the increased prevalence of synthetic opioid use and the high rates of polysubstance use. In this study, most opioid toxicity cases were successfully reversed prior to the ED arrival, with a substantial proportion managed by bystanders and first responders. These findings underscore the critical role of community access to naloxone and public education. Despite frequent ED contact following reversal, initiation of or referral for OAT was rare, highlighting a missed opportunity to engage patients in evidence-based treatment at a high-risk moment. Interventions to integrate addiction care pathways into post-reversal ED care are needed.
